# The impact of patent activity on idiosyncratic volatility in U.S. pharmaceutical companies

**DOI:** 10.1371/journal.pone.0334970

**Published:** 2025-10-27

**Authors:** Emre Atilgan, Fethiye Burcu Türkmen Ceylan, Ömer Tuğsal Doruk, Hasan Murat Ertuğrul, Ahmet Yasir Barak

**Affiliations:** 1 Department of Health Management, Trakya University, Edirne, Türkiye; 2 School of Global Development, University of East Anglia, Norwich, United Kingdom; 3 Department of Finance, Faculty of Economics and Administrative Sciences, Kırşehir Ahi Evran University, Kırşehir, Türkiye,; 4 Department of Economics, Kyrgyz-Turkish Manas University, Bishkek, Kyrgyzstan; 5 Department of Business Administration, Adana Alparslan Türkeş Science and Technology University, Adana, Türkiye; 6 Department of Economics, Anadolu University, Eskişehir, Türkiye; 7 Department of Accounting and Finance, Isparta Applied Sciences University, Isparta, Türkiye; Public Library of Science, UNITED KINGDOM OF GREAT BRITAIN AND NORTHERN IRELAND

## Abstract

This study examines the impact of patent activity on the idiosyncratic volatility (IVOL) of U.S. pharmaceutical companies, addressing a critical gap in the literature on the relationship between innovation and firm-specific risk. Using panel data from Thomson Reuters/Refinitiv covering 2,910 firms over 2005−2024, we employ the Fama-French 5-factor model to isolate firm-specific volatility and analyze how patent events and pharmaceutical development activities affect stock price risk. Our findings reveal a complex relationship between innovation and volatility that varies by development stage. While patent activity overall reduces idiosyncratic volatility, early and mid-stage development projects (Phase I and II) initially increase firm-specific risk, reflecting inherent uncertainties in drug development. Conversely, newly launched products significantly reduce volatility, indicating that risk mitigation occurs primarily at commercialization. These relationships remain robust during crisis periods, including the 2008−09 financial crisis and COVID-19 pandemic. The results provide valuable insights for investors seeking to assess pharmaceutical investment risks, managers optimizing innovation portfolios, and policymakers designing intellectual property frameworks. The study’s focus on the U.S. market and reliance on patent counts rather than quality measures suggest important avenues for future research across different regulatory environments and innovation metrics.

## Introduction

Patents play a pivotal role in the pharmaceutical industry, acting as powerful tools that not only safeguard innovation but also shape market competition and firm value. By granting exclusivity, patents protect the substantial R&D investments that companies make, allowing them to secure market positions, deter competitors, and capitalize on their innovations. This protective shield often translates into enhanced profitability and increased firm valuation, making patents essential strategic assets in the sector.

However, patents do more than just protect innovations; they also serve as key signals of a company’s technological prowess and growth potential. Investors and analysts closely track patent activity—such as filings, approvals, and citations—since these factors provide critical insights into a firm’s innovation trajectory and market stance. Such patent-related developments can drive shifts in market expectations and have a direct impact on stock prices. Yet, while patents are crucial for securing competitive advantage, they also introduce distinct financial complexities that can influence market dynamics.

In the pharmaceutical sector, characterized by high uncertainty and substantial costs, patent events often lead to significant stock price volatility. New patent approvals, expirations, or legal disputes can cause sudden changes in investor sentiment, driving fluctuations in a company’s specific risk profile—what we refer to as idiosyncratic volatility. Idiosyncratic volatility (IVOL) defines the volatility of the price of a particular stock due to conditions unique to the firm and not in the market at large [1]. These types of risks are not systematic risks, that is, they do not originate from market conditions of the economy but are the outcomes of financial status, managerial decisions, and competitive or operational effectiveness of the company. Volatility related to specific stocks specifically, is adversely associated with returns, underlining its importance as a risk factor to investors and firms [2]. High IVOL implies that investors have doubts or concerns about the solvency of a company and its future earnings generation capability and therefore can forecast expected returns well and investors demand a risk premium.

From the standpoint of efficient markets at the aggregate level, IVOL is likely to disrupt efficient capital allocation by diminishing investors’ confidence and liquidity [3]. The Efficient Market Hypothesis (EMH) suggests that stock prices reflect all available information and that excess returns cannot be consistently generated through market timing or stock selection [4]. However, idiosyncratic volatility introduces noise into this efficient pricing mechanism, potentially leading to mispricing and resource misallocation. In efficient markets, increased IVOL may indicate either genuine information flow about firm-specific developments (such as patent events) or noise that temporarily distorts proper asset valuation. When investors can accurately interpret patent-related information, market prices should efficiently adjust to reflect the economic value of these intellectual property assets.

In addition, innovation activities are central to IVOL. Though highly innovative ventures further volatility through risky research development projects and patenting processes, successful innovations may minimize future uncertainty and hence hurts IVOL [5].

Innovation is seen as a critical factor for firms to gain a competitive advantage and sustain long-term growth. Yet, the relationship between innovation and IVOL is still an object of discussion in the literature and both the positive and the negative effects of innovation on IVOL have been discussed and analyzed in a variety of works [5–7]. Hirshleifer, Hsu (5) proposed that although innovation can generate positive value for a firm, it also leads to an elevation in the level of idiosyncratic risk of the firm. Thus, there is an increase in uncertainty in the market due to new product and service developments and this can cause a fluctuation in the stock prices. As for IVOL, for the most part, useful innovations that may be protected through the patenting processes can lower the uncertainty concerning further money flows, decreasing IVOL. In a similar vein, Alshammasi and Almomen (6)established that innovation outputs, patents more specifically, decrease the idiosyncratic risks of firms. The findings have revealed that patents have far-reaching implications for firms with high information uncertainty by helping the firms cope with market risks.

While exploring the impact of R&D investments on IVOL, R&D expense reductions were most beneficial in lowering idiosyncratic risks for firms with a small capitalization and low leverage [8]. This means that the effect of innovation on IVOL is not only mediated by, for example, firm size or financial structure but it is also incorporated the developmental level of the market and the specific strategic choices of the firm. These findings are also further corroborated by Mazzucato and Tancioni (7) that revealed that while innovation tends to lead to volatility in the short run, innovation processes that have been successful in the long run have a risk reducing impact. It especially focuses on the fact that high IVOL in innovative firms might mean that there is a stronger feedback effect, which increases the efficiency of innovation related investments [9].

While previous studies have examined the relationship between innovation and stock market performance in various industries (e.g., [5,7]), the specific link between patent activity and idiosyncratic volatility in the pharmaceutical sector remains underexplored. For instance, studies by Alshammasi and Almomen (6) and Kumar and Li (9) have touched on innovation and firm-specific risk but did not focus on the unique dynamics of the pharmaceutical industry, where patent events create distinctive market reactions due to the industry’s high R&D intensity and regulatory hurdles.

The pharmaceutical sector provides an ideal context for this study due to its exceptional reliance on patent protection compared to other industries. Unlike sectors where alternative mechanisms such as trade secrets or first-mover advantages may suffice, pharmaceutical firms face particularly high R&D costs (averaging $2.6 billion per approved drug), lengthy development cycles (10–15 years), and ease of imitation once a compound is identified [10]. These characteristics make patent protection critically important and potentially more impactful on risk profiles than in other industries.

The U.S. market serves as an appropriate setting for this analysis because it represents the world’s largest pharmaceutical market, with robust patent enforcement mechanisms, transparent financial reporting requirements, and a well-established regulatory framework for drug approval. These institutional features allow for a clearer examination of the relationship between patent activities and market responses.

This study focuses on examining how patent activity influences the idiosyncratic volatility of pharmaceutical companies. It aims to delve into the extent to which patents drive stock price movements that are unique to the firm, providing insights into how the market responds to patent-related news in this highly competitive sector. Using a comprehensive panel data set derived from Thomson Reuters Eikon for patent information, the study employs econometric models, including the Fama-French 5-factor model, to quantify the effects of patent activity on idiosyncratic volatility. This methodological approach allows for a rigorous analysis of how different stages of drug development and patent milestones influence firm-specific risk.

This study is significant as it bridges a critical gap in our understanding of how patent activity influences firm-specific risk in the pharmaceutical industry. By examining the relationship between patent events and idiosyncratic volatility, this research provides valuable insights into the financial implications of innovation strategies. This is particularly relevant in the pharmaceutical sector, where substantial investments in R&D and the outcomes of patent-related activities can significantly impact a company’s market position and financial stability.

This paper makes several key contributions to the existing literature. It provides a comprehensive analysis of the impact of patent activity on idiosyncratic volatility in the pharmaceutical industry, an area that has been underexplored despite its importance. The study employs a novel approach by examining the effects of different stages of pharmaceutical development (Phase I, II, and III) on firm-specific risk, offering a more nuanced understanding of how the drug development process influences market perceptions and stock volatility.

Given the high uncertainty and regulatory challenges in the pharmaceutical sector, this study contributes to a deeper understanding of how innovation activities, particularly patenting, influence firm-specific financial risks. By linking patent dynamics to stock price volatility, the research not only fills a critical gap in the literature but also provides actionable insights that can guide decision-making in this high-stakes industry with several important policy implications. For policymakers, the results highlight the need for a balanced approach to patent protection that encourages innovation while also considering the potential impact on market stability and investor risk. The study’s insights into how different stages of drug development affect firm-specific risk could inform regulatory decisions regarding the disclosure requirements for pharmaceutical companies, potentially leading to more efficient market responses to R&D activities. For investors and financial regulators, the findings underscore the importance of considering patent-related events in assessing the risk profiles of pharmaceutical companies, which could lead to more informed investment decisions and improved risk management strategies.

This study addresses three key research questions: (1) How do patent approvals affect the idiosyncratic volatility of pharmaceutical firms? (2) Does the relationship between patent activity and idiosyncratic volatility differ across various stages of drug development (Phase I, II, and III)? and (3) What are the differential impacts of patent-related events, such as expirations and legal disputes, on firm-specific risk in this sector?

The remainder of this paper is structured to guide readers through our research process and findings. We begin with a comprehensive review of the relevant literature, developing our theoretical framework. This is followed by a detailed description of our data sources and methodology, including the construction of our idiosyncratic volatility measure and the econometric models employed. We then present our empirical results, encompassing the main findings on the relationship between patent activity and idiosyncratic volatility, as well as the impact of different pharmaceutical development phases. A discussion of the implications of our findings follows, addressing limitations and suggesting directions for future research. The paper concludes with a summary of our key findings and their broader implications for the pharmaceutical industry and financial markets.

### Theoretical background and literature review

The Efficient Market Hypothesis (EMH) serves as a fundamental theoretical framework for understanding how financial markets process and incorporate information into asset prices. Originally formulated by Fama (4), the EMH posits that asset prices fully reflect all available information, making it impossible to consistently achieve higher returns than average market returns on a risk-adjusted basis. This theory is particularly relevant to our study as it provides the theoretical foundation for understanding how patent-related information affects stock price volatility in pharmaceutical markets.

The EMH is categorized into three distinct forms, each differing in the scope of information considered to be reflected in stock prices [4]. The weak form efficiency suggests that all past trading information, including historical prices and volumes, is fully incorporated into current stock prices, rendering technical analysis ineffective for generating excess returns [11]. The semi-strong form efficiency extends this concept to include all publicly available information, such as financial statements, news releases, and patent announcements, implying that neither fundamental nor technical analysis can provide sustainable advantages [12]. The strong form efficiency represents the most comprehensive level, claiming that even private information is reflected in stock prices, though empirical evidence suggests that insiders can sometimes earn above-average returns, indicating markets may not be fully strong-form efficient [13].

In the context of pharmaceutical financial markets, the application of EMH becomes particularly complex due to the unique characteristics of innovation-related information. Research demonstrates that patent approvals, R&D announcements, and drug development news significantly influence stock price movements, reflecting the market’s sensitivity to innovation-related information [14]. Event studies have consistently shown that pharmaceutical firms experience abnormal returns following patent announcements and FDA approvals, suggesting that while markets generally react efficiently to new information, the magnitude and timing of these reactions can vary considerably. However, the EMH framework faces challenges in pharmaceutical markets, as evidenced by instances where market participants do not fully incorporate the implications of innovation capabilities into stock prices, particularly in industries with short time lags between technological advances and profit realization [15]. These deviations from perfect efficiency highlight the complex relationship between innovation activities and market dynamics, where factors such as information leakage, financing constraints, and the inherent uncertainty of pharmaceutical development can lead to market inefficiencies [16].

The pharmaceutical industry, characterized by high research and development (R&D) costs, long product development cycles, and significant regulatory hurdles, relies heavily on patents to protect innovations and secure competitive advantages. Patents play a crucial role in this sector by providing a period of market exclusivity that allows firms to recoup their substantial R&D investments [17,18]. This exclusivity period enables companies to earn monopolistic profits, offsetting the considerable expenditures incurred during the research and development phases [10,19].

The importance of patents in pharmaceuticals is particularly pronounced compared to other industries due to the ease with which new drugs can be imitated [10,20]. Patents serve as a primary mechanism for protecting innovative activities and fostering innovation in this sector [21]. Moreover, the disclosure of information through patents plays a significant role in shaping technological trajectories by enabling firms to monitor each other’s R&D efforts, thus avoiding redundant research and identifying promising research paths [22]. However, the effectiveness of the patent system in promoting innovation is debated. Some researchers suggest that a strong patent system may not always be beneficial, advocating for minimal levels of patent protection to foster competition and innovation [23,24]. This perspective highlights the complex relationship between patent protection and market dynamics in the pharmaceutical industry.

The institutional economics perspective, particularly drawing from Veblen’s work, provides valuable insights into the role of patents in the pharmaceutical industry. Patents serve not only as legal protections for innovation but also as critical financial assets that significantly impact firm valuation and market dynamics [25,26]. While not financial assets in the strict accounting sense (as defined in IAS 32), patents represent valuable intellectual property resources that generate future economic benefits and influence financial performance. In the context of financialized business models, patents act as strategic assets driving the economic value of pharmaceutical companies. Veblen emphasized that intangible assets like patents provide firms with monopolistic power, enabling them to extract economic rents not directly tied to productive processes [26]. This financialization is evident as patents influence key performance indicators such as Earnings Per Share (EPS), which often show significant improvement following patent approval [27]. The impact of patents extends beyond individual firms to shape broader market dynamics, particularly during crises. Government support in the form of grants and subsidies often accelerates the development of essential drugs, aligning with Mazzucato [28] concept of the “entrepreneurial state”. However, this public funding combined with private patent rights can amplify market dominance, leading to monopolistic pricing and potentially limiting access to essential medications [27,29]. The ethical implications of patents in pharmaceuticals become particularly acute during crises, where the need for timely access to critical medicines often conflicts with profit motives. Veblen’s framework emphasizes that intangible assets like patents often serve financial extraction rather than productive or societal utility [25,26]. This raises fundamental questions about balancing innovation incentives with the imperative to make life-saving drugs accessible, especially when public funds are involved in their development.

Patents significantly impact firm performance and market dynamics through various mechanisms. They can enhance a firm’s customer capital by improving customer perceptions of product novelty and quality, positively impacting firm performance and financial market valuation [30]. Firms holding academic patents benefit from knowledge spillovers, which enhance their market power, although they may experience a negative impact on short-term profitability due to the high costs associated with acquiring and exploiting these patents [31]. In the context of strategic competition, firms engaging in patent litigation or other aggressive patent strategies tend to perform better, especially when they have a diversified technological portfolio. However, the benefits of such strategies can be mitigated by open innovation practices [32]. Firms with larger market shares are more likely to exploit patents through various modes, including own use, licensing, and blocking, with the blocking strategy strongly associated with maintaining competitive advantage [33].

The commercialization performance of patents is significantly influenced by a firm’s complementary assets and the appropriability of its innovations, with market sensing capabilities further enhancing this relationship [34]. The propensity to trade patents is influenced by the alignment of a patent’s technology structure with potential buyers’ portfolios, although this propensity is reduced when there is significant product-market overlap or when the original assignee has superior technological capabilities [35].

The relationship between patents and financial risk is multifaceted, involving both the potential for patents to mitigate financial risk and the risks associated with leveraging patents in financial strategies. Patents can enhance a firm’s resilience during financial crises by providing strategic resources that help sustain competitive advantages [36]. They also serve as a quality signal to external investors, potentially mitigating internal liquidity constraints, particularly beneficial for smaller firms [37–39].

The size of a company’s patent portfolio has been shown to positively correlate with its credit rating, suggesting that patents can enhance a firm’s debt capacity by serving as valuable assets that improve creditworthiness [40]. However, patents with high forward citations may negatively impact credit ratings due to the potential for patent lawsuits, which pose financial risks to creditors [40,41].

The use of patents as collateral in financial transactions, as seen in patent pledge policies, can increase the risk of stock price crashes, particularly for firms with strong financial standing and excessive managerial confidence [42]. Firms with higher idiosyncratic asset volatility often have more dispersed debt maturity structures, a strategy to manage rollover risk, which is particularly important for pharmaceutical firms that rely heavily on external financing for R&D activities [43].

Patent approvals, especially those that are considered radical, tend to lead to positive stock market reactions. This is because radical patents often signify breakthrough innovations that can potentially lead to significant competitive advantages and future revenue streams for the company [44]. News related to acquisitions and clinical trials can lead to significant abnormal returns [45,46]. Patent disputes and litigation events can have profound impacts on stock prices, with firms in a strong position in patent lawsuits experiencing positive stock returns, while those in a weaker position face negative returns [47,48].

The mere filing of a patent opposition can result in statistically significant negative abnormal returns, highlighting the market’s sensitivity to potential patent invalidation [49]. Patent expirations can lead to increased competition and reduced market exclusivity, potentially impacting stock prices negatively and often leading to increased volatility as firms approach patent cliff events [18,50].

Firm-specific risk known as idiosyncratic volatility reflects the part of a stock’s price variation coming from company-specific elements including financial construction and managerial choices as well as operational success outside market influences [1]. Firm risk which remains independent from economic conditions receives a separate classification from systematic risk and serves as a business variable that reveals corporate activities and production uncertainties. Researchers have explored the information embedded within IVOL because it reveals undisclosed firm details that traditional risk modeling frameworks cannot detect. When the level of IVOL reaches high levels, it demonstrates financial and earnings uncertainties which diminish investor faith thus reducing market liquidity and capital allocation system efficiency [3].

Empirical research reveals additional findings about how idiosyncratic volatility develops as well as its component sources. Multiple research studies showed how firm-level volatility increased consistently with market volatility during multiple decades while stock correlations diminished, and market models decreased in effectiveness [1]. Using the Fama-French three-factor model Xu and Malkiel (3) established the upward movement and linked it to two variables: growing institutional ownership together with market expectations of profit growth. Bekaert, Hodrick [51] studied global idiosyncratic volatility from a cyclical and macroeconomic viewpoint which showed that the factor behaves with mean reversion and shows temporary spikes instead of enduring trends. Measurement of idiosyncratic volatility typically involves asset-pricing models, residual variance analysis, GARCH models, and rolling window estimations [52–55].

Investors in the pharmaceutical industry perceive and react to patent news with significant interest. Patent approvals, strategic alliances involving patent sharing, and R&D investments accompanied by new product announcements generally lead to positive market reactions [46,56]. Conversely, patent expirations, litigation announcements, and patent challenges often result in negative market reactions [18,47,49].

The study of patents and volatility employs a variety of methodological approaches, including econometric models, network-based approaches, text mining techniques, event studies, panel data analysis, instrumental variable approaches, and agent-based modeling [24,30,45,57–60]. These diverse approaches reflect the complexity of studying the relationship between patents and volatility, each offering unique insights into different aspects of this relationship.

The pricing of idiosyncratic volatility remains a complex puzzle, with existing explanations based on investor sentiment and opinion differences proving inadequate. Researchers such as Zaremba, Czapkiewicz (61) and Chen, Wang (55) highlight these challenges, suggesting that traditional risk-return frameworks may not fully capture the complexities of firm-specific risk in innovation-intensive industries like pharmaceuticals.

The relationship between idiosyncratic volatility and stock returns is non-linear and significantly affected by financial crises, indicating the need for models that can capture these dynamics across different market conditions [55]. Some researchers argue that the observed risk-return relationship could be a result of statistical artifacts rather than real economic factors, challenging the notion of a true anomaly in idiosyncratic volatility pricing [61].

The literature demonstrates various conflicting and detailed findings about how innovation relates to idiosyncratic volatility in driving firm competitiveness and long-term value creation. Innovation processes and patentable inventions serve to decrease market uncertainty over future cash flows because they establish valuable intangible company assets while diminishing market information asymmetry [6]. Organizations facing significant information uncertainty attain the most prominent protection against market-related risks through their innovation activities. Stock price volatility tends to increase because of unpredictable outcomes that result from innovative processes which happen especially during initial phases where new products and unproven technologies are being developed [5]. Mazzucato and Tancioni (60) state that short-term innovation-related volatility matches long-run stabilization of risk by sustaining successful innovation initiatives. High idiosyncratic volatility in innovative firms indicates efficient feedback loops and improved innovation investment efficiency beyond risk measurement [9], especially when operating in pharmaceuticals which demand innovative approaches to deliver successful performance.

The relationship between patents and idiosyncratic volatility in the pharmaceutical sector is complex. R&D-intensive firms, which are likely to hold more patents, experience higher volatility in returns, suggesting that the uncertainty associated with future profits from innovations contributes to increased idiosyncratic risk [60]. Firms that deviate strategically from industry norms, possibly through unique patent strategies, tend to exhibit higher idiosyncratic return volatility [62].

Increased competition tends to raise idiosyncratic volatility relative to systematic volatility, as firms face more firm-specific cost shocks that cannot be easily passed on to consumers [63]. The complexity of patent thickets can result in more frequent litigation, further influencing stock price volatility and investor perceptions [64]. Variations in patent granting probabilities and examination durations across countries can affect firms’ strategic decisions and their subsequent market performance, potentially impacting idiosyncratic volatility [65].

Despite the extensive research in this field, several gaps and limitations exist in the current literature on patents and idiosyncratic volatility. Many studies on idiosyncratic volatility do not incorporate direct measures of innovation, such as patent data, which limits the understanding of how technological advancements impact firm-specific risks.

Future research could explore the impact of emerging technologies, such as AI-enabled digital identity systems, on patent valuation and associated volatility. Additionally, investigating the role of patents in open ecosystems and their implications for policymakers could provide valuable insights. As the pharmaceutical industry faces ongoing pressures to innovate and manage risks, understanding the role of patents in shaping firm-specific volatility will remain a critical area of research and practice.

Based on the theoretical framework and literature review presented above, we develop the following research hypotheses:

Hypothesis 1: Patent activity is negatively associated with idiosyncratic volatility in pharmaceutical firms. This hypothesis is grounded in the notion that patents reduce market uncertainty by securing exclusive rights to commercialize innovations, thereby providing clearer expectations about future cash flows. Alshammasi and Almomen (6) suggests that successful innovation outputs like patents can decrease firm-specific risks by reducing information uncertainty.

Hypothesis 2: The impact of pharmaceutical development projects on idiosyncratic volatility differs by development phase, with more advanced phases (Phase III) having a stronger negative association with volatility than earlier phases (Phase I and II). This hypothesis is based on the progressive reduction of uncertainty as drug candidates advance through clinical trials. Early-stage projects carry higher technical and regulatory risks, while late-stage projects that have passed critical safety and efficacy evaluations represent lower risks and greater proximity to potential market approval and commercialization, as discussed in the research by Mazzucato and Tancioni (60).

## Dataset and methodology

This study utilizes Thomson Reuters Eikon database (now part of the London Stock Exchange Group – LSEG), data source to examine the dynamics of pharmaceutical companies. This comprehensive financial dataset serves as the foundation for calculating the idiosyncratic volatility of the sample firms, enabling an in- depth analysis of their market performance and associated risk factors. The database provides detailed firm-level patent information and data on pharmaceutical projects across various development stages, including Phase I, Phase II, and Phase III. This information facilitates the examination of innovation activities and project progression within the pharmaceutical sector. Access to the Thomson Reuters Eikon database is available through a subscription to the Eikon Refinitiv platform [Eikon Refinitiv platform is accessible via www.eikon.refinitiv.com].

The statistical analyses in this study were conducted using Stata version 17, which facilitated data management and the implementation of econometric models, including the Fama-French 5-factor model and panel regressions.

The Fama-French five-factor model is commonly employed in the calculation of idiosyncratic volatility, as it comprehensively captures risks associated with the market. Accordingly, in the first stage of the analysis, the five-factor Fama-French model was estimated using monthly data spanning from January 2005 to December 2024, resulting in a sample of 355,544 observations across 2,910 companies, with an average data coverage of 122.18 months per company. Differenced natural logarithm price data were utilized in the estimation. The selection of companies was based on the North American Industry Classification System (NAICS). Since the dependent variable is specified in first differences, it does not exhibit a unit root. However, all additional variables included in the analysis, except the dummy variable, were subjected to panel unit root tests (specifically, the Panel ADF Fisher-type unit root test), and all were found to be stationary.

The U.S. pharmaceutical industry represents an ideal context for examining the relationship between patents and idiosyncratic volatility due to its unique characteristics. During our sample period (2005–2024), this sector experienced significant transformations, including waves of consolidation, increasing R&D intensity, and evolving regulatory frameworks. The industry is characterized by high R&D intensity, with pharmaceutical firms in our sample investing an average of 20.6% of their revenues in research and development, compared to approximately 3.5% across all industries in the S&P 500. The drug development process is notably long and expensive, with estimates suggesting an average cost of $2.6 billion and 10–15 years from discovery to market [66].

The regulatory environment during this period saw important changes, including the implementation of the Prescription Drug User Fee Act (PDUFA) VI in 2017, which aimed to accelerate approval times, and the 2010 Affordable Care Act, which expanded market access but introduced new industry fees. These regulatory shifts potentially influenced patent values and market perceptions.

The measurement of idiosyncratic volatility (IVOL) is grounded in both asset pricing theory and the Efficient Market Hypothesis framework. Under EMH, systematic risk factors captured by asset pricing models should be efficiently incorporated into stock prices, leaving only genuine firm-specific uncertainty in the residuals. Following established literature [1,2], we employ the Fama-French 5-factor model to isolate this firm-specific component of volatility. This model is theoretically superior to single-factor models as it accounts for multiple systematic risk factors (market, size, value, profitability, and investment) that EMH suggests should be efficiently priced by markets [67]. The residual volatility from this model represents the component of stock price movements that markets have not yet fully resolved through efficient information processing—precisely the component that patent events should theoretically affect by providing new information about firm prospects and reducing information uncertainty.

The Fama-French 5-factor model [67] extends the traditional 3-factor model by adding profitability (RMW) and investment (CMA) factors to the original market (MKT), size (SMB), and value (HML) factors. We employ this model because it provides a more comprehensive account of systematic risk factors, allowing for more accurate isolation of the truly idiosyncratic component of stock returns. The 5-factor model has been shown to explain approximately 71–94% of cross-sectional variation in expected returns [67], compared to 60–83% for the 3-factor model, resulting in more precise measurement of idiosyncratic volatility.

The model takes the form in Equation (1):


$$R_{i,t}-R_{f,t}=\alpha_{i}+\beta_{\text{1},i}\left(R_{m,t}-R_{f,t}\right)+\beta_{\text{2},i}SMB_{t}+\beta_{\text{3},i}HML_{t}+\beta_{\text{4},i}RMW_{t}+\beta_{\text{5},i}CMA_{t}+\varepsilon_{i,t}$$ (1)

Where, $R_{i,t}$ is the return on stock i on day t, $R_{f,t}$ is the risk-free rate on day t, $R_{m,t}$ is the market return on day t, $SMB_{t}$ (Small Minus Big) is the size factor, $HML_{t}$ (High Minus Low) is the value factor, $RMW_{t}$ (Robust Minus Weak) is the profitability factor, $CMA_{t}$ (Conservative Minus Aggressive) is the investment factor and $\varepsilon_{i,t}$ is the residual, representing the idiosyncratic return component.

In the initial stage of the analysis, the five-factor Fama-French model was estimated using monthly data from January 2005 to December 2024, as previously described, encompassing 355,544 observations and 2,910 companies with an average of 122.18 months per company. Returns were calculated using the differenced natural logarithm of price data for several methodological reasons. First, log transformations normalize the distribution of returns, thereby improving the statistical properties of the model. Second, log returns are time-additive, which is advantageous for multi-period analysis. Third, this approach is standard in financial econometrics and facilitates comparability with prior research [1,2].

The Fama-French 5-factor model regression, which includes market, size, profitability, value, and investment factors, as represented in Equation (2).


$$r_{i,t}=\alpha_{i}+\beta_{\text{1}}{\text{MKT}}_{t}+\beta_{\text{2}}{\text{SMB}}_{t}+\beta_{\text{3}}{\text{HML}}_{t}+\beta_{\text{4}}{\text{RMW}}_{t}+\beta_{\text{5}}{\text{CMA}}_{t}+\varepsilon_{i,t}$$ (2)

In this model, MKT represents the market factor, SMB is the size factor, RMW denotes the profitability factor, HML is the value factor, and CMA is the investment factor. The Fama- French daily data were sourced from Kenneth French’s website. The residuals from this regression are isolated for each firm, and their standard deviations are annualized to derive the firm’s idiosyncratic volatility. The monthly residuals were merged with the annual patent and R&D phase level information to form an annual data set. Therefore, the IVOL data, which was finally created between 2005–2024, was sampled for the years 2005–2024 after this merge process. The sample level considered in the study is between the years 2005–2024 selected according to the firm level patent information. Due to the availability of patent information and R&D projects in phase I, phase II and phase III, in pre-registration phase, and launching phase, the start and end dates of the sample were selected between these dates.

The classification of R&D projects is as follows: Phase I at the commencement of the process, Phase II upon completion of Phase I, and Phase III upon completion of Phase II and transition to Phase III. These phases may be conceptualized as research and development projects, which, as the number of successfully completed phases increases, approach completion. The main regression model linking idiosyncratic volatility (IVOL) with patent activity is specified in Equation 3:


$${\text{IVOL}}_{i,t}=\beta_{\text{0}}+\beta_{\text{1}}\text{PATEN}{\text{T}}_{i,t}+\beta_{n}X_{n,i,t}+\gamma +\varepsilon_{i,t}$$ (3)

Here, IVOL represents the idiosyncratic volatility, and PATENT stands for the change in the number of patents obtained annually. The matrix (X) includes control variables such as firm size (SIZE) and firm age (AGE).

Our selection of control variables follows established literature on the determinants of idiosyncratic volatility. Firm size (SIZE), measured as the natural logarithm of total assets, is included because larger firms typically exhibit lower volatility due to their diversified revenue streams, greater resource access, and higher analyst coverage [68,69].

Firm age (AGE), measured as years since founding, accounts for the reduced uncertainty that typically comes with operational experience and market establishment. Younger firms generally face higher uncertainty regarding their business models and growth trajectories, leading to greater stock price volatility [6,69–72].

To mitigate potential multicollinearity concerns that may result from high correlations among drug patent phases, separate models were estimated for each phase. The annualized idiosyncratic volatility is further analyzed in relation to pharmaceutical project using the following regression models:


$${\text{IVOL}}_{i,t}=\beta_{\text{0}}+\beta_{\text{1}}\text{PHASE}{\text{I}}_{i,t}+\beta_{n}X_{n,i,t}+\gamma +\varepsilon_{i,t}$$ (4)

$${\text{IVOL}}_{i,t}=\beta_{\text{0}}+\beta_{\text{1}}\text{PHASE}{\text{II}}_{i,t}+\beta_{n}X_{n,i,t}+\gamma +\varepsilon_{i,t}$$ (5)

$${\text{IVOL}}_{i,t}=\beta_{\text{0}}+\beta_{\text{1}}\text{PHASE}{\text{III}}_{i,t}+\beta_{n}X_{n,i,t}+\gamma +\varepsilon_{i,t}$$ (6)

$${\text{IVOL}}_{i,t}=\beta_{\text{0}}+\beta_{\text{1}}{PRE-REG}_{i,t}+\beta_{n}X_{n,i,t}+\gamma +\varepsilon_{i,t}$$ (7)

$${\text{IVOL}}_{i,t}=\beta_{\text{0}}+\beta_{\text{1}}{NEWLAUCH}_{i,t}+\beta_{n}X_{n,i,t}+\gamma +\varepsilon_{i,t}$$ (8)

In Equations (4–8), PHASE I, PHASE II, and PHASE III represent projects in their respective phases of development. PRE-REG is the products in the pre-registration phases, and NEWLAUNCH is the newly launched products.

Pharmaceutical development follows a standardized, sequential clinical trial process that is highly regulated by the FDA:

Phase I trials represent the initial stage of clinical testing, focusing primarily on safety. These trials typically involve 20–100 healthy volunteers or patients and assess the drug’s safety profile, appropriate dosage ranges, and pharmacokinetics. Phase I trials have the highest failure rate (approximately 40%) and represent the earliest stage of human testing.

Phase II trials involve larger groups of patients (100–500) who have the condition the drug is designed to treat. These trials assess both efficacy and side effects, determining whether the drug shows sufficient therapeutic effect to justify further testing. Phase II trials have a failure rate of approximately 30% and represent medium-risk development activities.

Phase III trials are large-scale studies (1,000–5,000 patients) that confirm effectiveness, monitor side effects, and compare the drug to commonly used treatments. These trials provide the primary evidence for FDA approval and market entry. With a failure rate of approximately 15%, Phase III trials represent the most advanced, lowest-risk stage of development before potential market entry.

PRE-REG (Pre-Registration) is the products that have completed clinical trials and are currently undergoing regulatory review by health authorities (FDA, EMA, etc.) for market approval. This phase involves regulatory agencies evaluating clinical data and safety profiles before granting authorization to sell the product.

NEWLAUNCH (Newly Launched Products) is the products that have received regulatory approval and been introduced to the market within a recent timeframe (typically 1–3 years). These products are in their initial commercialization phase, focusing on market penetration and establishing market share while continuing post-market monitoring.

The progression between the phases represents a substantial de-risking of the development process, with increasing certainty about both safety and efficacy. This sequential risk reduction forms the basis for our hypothesis regarding the differential impact of development phases on idiosyncratic volatility.

Descriptive statistics of the variables are shown in Table 1, highlighting the heterogeneity of the sample in terms of patent activity and idiosyncratic volatility among U.S. pharmaceutical firms. Table 2 presents the correlation matrix, indicating that multicollinearity is not a concern, except among Phase I, Phase II, and Phase III variables.

**Table 1:** Descriptive statistics.

*Variable*	*Mean*	*Std. dev.*	*Min*	*Max*	*Observations*
*IVOL*_*i,t*_	*.1565897*	*.1275129*	*.0016391*	*2.307.015*	*N = 19513*
					*n = 1727*
					*T-bar = 11.2988*
*PhaseI*_*i,t*_	*4.775.697*	*111.401*	*0*	*243*	*N = 2189*
					*n = 397*
					*T-bar = 5.51385*
*PhaseII*_*i,t*_	*4.268.719*	*8.292.216*	*0*	*123*	*N = 2631*
					*n = 426*
					*T-bar = 6.17606*
*PhaseIII*_*i,t*_	*3.778.374*	*6.290.558*	*0*	*84*	*N = 2608*
					*n = 407*
					*T-bar = 6.40786*
*SIZE*_*i,t*_	*1.811.838*	*2.698.583*	*2.302.585*	*2.614.602*	*N = 23298*
					*n = 1769*
					*T-bar = 13.1702*
*Age*_*i,t*_	*2.321.468*	*1.949.989*	*0*	*137*	*N = 22499*
					*n = 1751*
					*T-bar = 12.8492*
*Patents*_*i,t*_	*4.114.275*	*3.268.037*	*−10900*	*1067*	*N = 2564*
					*n = 421*
					*T-bar = 6.09026*
*Prereg*_*i,t*_	*1.982.612*	*7.200.826*	*1*	*857*	*N = 1179*
					*n = 276*
					*T-bar = 4.27174*
*Launched*_*i,t*_	*1.443.676*	*3.171.389*	*0*	*273*	*N = 1605*
					*n = 351*
					*T-bar = 4.57365*

**Table 2:** Correlation analysis.

*Variables*	*(1)*	*(2)*	*(3)*	*(4)*	*(5)*	*(6)*	*(7)*	*(8)*	*(9)*
*(1) IVOL* *i,t*	*1.000*								
*(2) Patent*_*i,t*_	*0.028*	*1.000*							
*(3) Age*_*i,t*_	−0.240*	−0.083*	*1.000*						
*(4) SIZE*_*i,t*_	−0.428*	−0.089*	0.319*	*1.000*					
*(5) PhaseI*_*i,t*_	−0.122*	*0.063*	0.062*	0.383*	*1.000*				
*(6) PhaseII*_*i,t*_	−0.178*	−0.130*	0.081*	0.444*	0.792*	*1.000*			
*(7) PhaseIII*_*i,t*_	−0.191*	−0.122*	0.113*	0.491*	0.710*	0.807*	*1.000*		
*(8) Prereg*_*i,t*_	*−0.007*	*0.021*	−0.065*	*0.012*	*−0.041*	*−0.023*	*−0.007*	*1.000*	
*(9) Launched*_*i,t*_	−0.067*	*0.015*	−0.058*	0.088*	*0.036*	*0.008*	*0.038*	0.345*	*1.000*

Note: *** p < 0.01, ** p < 0.05, * p < 0.1.

## Results

Table 1 presents the descriptive statistics for our sample of U.S. pharmaceutical firms over the period 2005–2024. The sample exhibits substantial heterogeneity across key dimensions, reflecting the diverse nature of the pharmaceutical industry ecosystem. Our primary dependent variable, idiosyncratic volatility (IVOL), displays a mean of 0.157 with a standard deviation of 0.128, indicating considerable variation in firm-specific risk across the sector. The distribution ranges from highly stable firms (minimum IVOL of 0.002) to those experiencing extreme volatility (maximum of 2.31), suggesting that pharmaceutical companies operate under markedly different risk profiles.

The innovation activity measures reveal the R&D-intensive nature of our sample firms. Patent activity shows significant dispersion (mean = 4.11, standard deviation = 32.68), with some firms maintaining minimal patent portfolios while others demonstrate extensive intellectual property generation. This heterogeneity is particularly pronounced in pharmaceutical development pipeline data: Phase I projects average 4.78 per firm but with substantial variation (standard deviation = 11.14), while Phase III projects, representing more mature development efforts, average 3.78 per firm with somewhat less dispersion (standard deviation = 6.29). This pattern is consistent with the industry’s funnel-shaped development process, where many early-stage projects are winnowed to fewer late-stage candidates.

The control variables exhibit expected characteristics for pharmaceutical firms. Firm size (SIZE) shows considerable variation (mean = 18.12, standard deviation = 2.70), encompassing both large multinational pharmaceutical companies and smaller biotech firms. Firm age similarly spans a wide range (mean = 23.21 years, standard deviation = 19.50), reflecting the industry’s mixture of established players and newer entrants focused on specialized therapeutic areas.

The correlation matrix in Table 2 provides initial insights into the relationships among our key variables. Most notably, patent activity exhibits a small positive correlation with idiosyncratic volatility (r = 0.028), which contrasts with our theoretical prediction but warrants careful interpretation in the multivariate context. This preliminary finding may reflect the complex dual nature of innovation in pharmaceuticals—while patents ultimately reduce risk, the innovation process itself may initially introduce uncertainty.

The pharmaceutical development phase variables show the expected negative correlations with IVOL, ranging from −0.122 (Phase I) to −0.191 (Phase III). This gradient aligns with our theoretical framework, where more advanced development stages should provide greater risk reduction. The high correlations among development phases (0.71–0.81) confirm our methodological decision to estimate separate models for each phase to avoid multicollinearity concerns.

Control variables behave as expected from prior literature. Both firm size (r = −0.428) and age (r = −0.240) show negative correlations with volatility, consistent with the stabilizing effects of scale and operational maturity in reducing firm-specific uncertainty.

Table 3 presents our main regression results testing the relationship between patent activity and idiosyncratic volatility. All models employ standard errors that are robust to both heteroscedasticity and autocorrelation, addressing the potential presence of these issues commonly observed in panel data due to substantial cross-sectional heterogeneity. The results provide mixed support for our theoretical predictions and reveal important nuances in how different aspects of pharmaceutical innovation affect firm-specific risk.

**Table 3:** Regression results.

	*(1)*	*(2)*	*(3)*	*(4)*	*(5)*	*(6)*
	*IVOL*_*i,t*_	*IVOL*_*i,t*_	*IVOL*_*i,t*_	*IVOL*_*i,t*_	*IVOL*_*i,t*_	*IVOL*_*i,t*_
*Age*_*i,t*_	−0.000482^**^ (−9.30)^*^	−0.000443^***^ *(−9.52)*	*−0.000521* ^***^ *(−10.62)*	−0.000690^**^ (−9.24)^*^	−0.000430^***^ *(−9.11)*	−0.000258^***^ *(−4.59)*
*Size*_*i,t*_	−0.0167^***^ *(−21.49)*	−0.0159^***^ *(−22.29)*	−0.0166^***^ *(−20.10)*	−0.0158^***^ *(−16.27)*	−0.0109^***^ *(−13.88)*	−0.0142^***^ *(−13.63)*
*PhaseI*_*i,t*_	0.000409^***^ *(5.50)*					
*PhaseII*_*i,t*_		0.000282^*^ *(2.43)*				
*PhaseIII*_*i,t*_			0.000338^*^ *(2.09)*			
*Patents*_*i,t*_				−0.00000407^**^ *(−3.14)*		
*Launched*_*i,t*_					−0.0000867^*^ *(−2.05)*	
*Prereg*_*i,t*_						*−0.00000380 (−0.16)*
*Year dummies*	*Yes*	*Yes*	*Yes*	*Yes*	*Yes*	*Yes*
*β*_*0*_	0.451^***^ *(20.32)*	0.427^***^ *(22.88)*	0.442^***^ *(21.93)*	0.446^***^ *(20.17)*	0.346^***^ *(16.87)*	0.391^***^ *(16.97)*
*N*	*2096*	*2484*	*2462*	*2487*	*1540*	*1115*
*R* ^*2*^	*0.267*	*0.267*	*0.254*	*0.194*	*0.218*	*0.220*

*Note: t statistics in parentheses.* ^*^ *p < 0.05,* ^**^ *p < 0.01,* ^***^ *p < 0.001. Heteroscedasticity and autocorrelation robust standard errors are employed (see Appendix C).*

Contrary to Hypothesis 1, we find that patent grants are associated with a small but statistically significant reduction in idiosyncratic volatility (β = −0.00000407, t = −3.14, p < 0.01). While the coefficient’s magnitude appears modest, the economic interpretation requires careful consideration. Given the annual nature of our patent data and the substantial variation in patent activity across firms, this effect represents a meaningful risk reduction for firms with active patent portfolios. For a firm experiencing a one-standard-deviation increase in patent activity (approximately 33 patents), the model predicts a reduction in annualized IVOL of approximately 0.00013, representing about 0.08% of the sample mean volatility.

The pharmaceutical development phase results provide strong support for Hypothesis 2, revealing that the relationship between innovation and risk varies significantly across development stages. Phase I projects show a positive association with volatility (β = 0.000409, t = 5.50, p < 0.01), consistent with the high uncertainty inherent in early-stage drug development. Phase II projects maintain a smaller but significant positive effect (β = 0.000282, t = 2.43, p < 0.05), while Phase III projects show a positive coefficient (β = 0.000338, t = 2.09, p < 0.05).

These findings suggest that ongoing development activities, regardless of stage, introduce some degree of firm-specific uncertainty, likely reflecting the substantial resource commitments and uncertain outcomes associated with pharmaceutical R&D. However, the gradient across phases is less pronounced than hypothesized, indicating that risk reduction primarily occurs at the commercialization stage rather than during development.

The analysis of post-development activities provides the strongest support for our risk-reduction hypothesis. Newly launched products demonstrate a significant negative relationship with volatility (β = −0.000867, t = −2.05, p < 0.05), indicating that successful market entry substantially reduces firm-specific risk. This effect aligns with our theoretical framework, where the transition from uncertain development outcomes to realized market performance reduces information asymmetry and stabilizes investor expectations.

Pre-registration products show a negative but statistically insignificant effect (β = −0.000380, t = −0.16), suggesting that regulatory approval processes may not provide sufficient certainty to meaningfully reduce volatility until actual market launch occurs.

The control variables perform consistently with expectations across all model specifications. Firm size exhibits a strong negative relationship with volatility (coefficients ranging from −0.0109 to −0.0167, all significant at p < 0.01), confirming that larger firms experience lower idiosyncratic risk. Firm age shows a consistently negative and significant relationship with volatility across most models, supporting the hypothesis that operational maturity reduces firm-specific uncertainty.

The explanatory power of our models, while modest (R² ranging from 0.154 to 0.267), is consistent with prior studies of idiosyncratic volatility in innovation-intensive industries. The relatively low R² values reflect the inherently noisy nature of firm-specific volatility and suggest that patent-related factors, while statistically significant, represent only one component of the complex risk environment facing pharmaceutical firms.

To validate the temporal stability of our findings, we conduct subsample analyses during two critical stress periods: the 2008–09 global financial crisis and the COVID-19 pandemic (2020–2021). These analyses, presented in Table 4, are theoretically important because crisis conditions typically amplify volatility and may alter how markets process patent-related information.

**Table 4:** Crisis and COVID-prone idiosyncratic volatility.

	(1)	(2)	(3)	(4)	(5)	(6)
	IVOL_i,t_	IVOL_i,t_	IVOL_i,t_	IVOL_i,t_	IVOL_i,t_	IVOL_i,t_
**Panel A: 2008−09 Financial Crisis**						
phaseI	0.000989^*^ (2.25)					
age	0.000207 (0.85)	−0.000406 (−1.14)	−0.000761 (−1.91)	−0.00101^*^ (−2.50)	0.000375 (1.18)	−0.000279 (−1.38)
size	−0.0239^***^ (−6.38)	−0.0208^***^ (−5.93)	−0.0246^***^ (−6.26)	−0.0191^*^(−2.57)	−0.0135^***^ (−5.16)	−0.0147^***^ (−6.95)
phaseII		0.0000421 (0.06)				
phaseIII			0.00113 (1.21)			
patents				0.0000683 (0.56)		
prereg					0.0000668 (0.94)	
Time fixed effects	Yes	Yes	Yes	Yes	Yes	Yes
β_0_	0.624^***^ (8.14)	0.594^***^ (8.76)	0.676^***^ (8.28)	0.559^***^ (3.88)	0.421^***^ (7.78)	0.446^***^ (9.94)
*N*	78	97	91	72	68	107
*R* ^2^	0.433	0.310	0.257	0.185	0.346	0.291
F stat., p-val.	0.00	0.00	0.00	0.00	0.00	0.00
**Panel B: COVID periods**						
	(1)	(2)	(3)	(4)	(5)	(6)
	IVOL_i,t_	IVOL_i,t_	IVOL_i,t_	IVOL_i,t_	IVOL_i,t_	IVOL_i,t_
phaseI	0.0000899 (0.43)					
Age	−0.000413^**^ (−2.82)	−0.000284^*^ (−2.31)	−0.000536^***^ (−3.34)	−0.000463^*^ (−2.41)	−0.000142 (−0.68)	−0.000671^***^(−5.37)
Size	−0.0148^***^ (−7.14)	−0.0137^***^ (−7.77)	−0.0129^***^ (−6.22)	−0.0134^***^ (−5.91)	−0.0150^**^ (−2.82)	−0.0105^***^ (−4.96)
phaseii		0.0000381 (0.17)				
phaseiii			−0.000427 (−0.81)			
patents				−0.0000717 (−1.60)		
Prereg					0.000440 (0.84)	
launched						−0.0000583
Time fixed effects	Yes	Yes	Yes	Yes	Yes	(−0.44) Yes
β_0_	0.470^***^ (10.97)	0.435^***^ (12.14)	0.435^***^ (10.66)	0.441^***^ (9.79)	0.450^***^(4.00)	0.360^***^ (8.09)
*N*	412	449	450	471	166	230
*R* ^2^	0.188	0.182	0.154	0.103	0.120	0.191
F stat., p-val.	0.00	0.00	0.00	0.00	0.00	0.00

*Note: t* statistics in parentheses. ^*^ *p* < 0.05, ^**^ *p* < 0.01, ^***^ *p* < 0.001. Heteroscedasticity and autocorrelation robust standard errors are employed. *Note: t* statistics in parentheses. ^*^ *p* < 0.05, ^**^ *p* < 0.01, ^***^ *p* < 0.001. Heteroscedasticity and autocorrelation robust standard errors are employed.

During the financial crisis, the core relationships maintain their statistical significance and directional consistency. Notably, the risk-reducing effects of advanced-stage projects appear amplified during this period, suggesting that investors place premium value on the certainty provided by late-stage developments when market-wide uncertainty is elevated. The persistence of patent effects during a period of general market distress supports our interpretation that these relationships reflect fundamental economic mechanisms rather than period-specific sentiment.

The pandemic period provides a particularly stringent test of our hypotheses, given the unprecedented focus on pharmaceutical innovation and accelerated regulatory processes. Our key findings remain robust during this period, with patent activity continuing to demonstrate risk-reducing effects even amid dramatically altered industry dynamics. This stability reinforces our conclusion that patents serve as fundamental risk-mitigating assets rather than merely reflecting favorable market sentiment toward pharmaceutical innovation.

The consistency of results across these diverse market conditions strengthens confidence in our main conclusions and suggests that the patent-volatility relationship reflects persistent structural features of the pharmaceutical industry rather than time-specific or market-condition-dependent phenomena.

To ensure our findings are not driven by omitted variable bias, we conducted additional robustness checks incorporating alternative control variables (X, Y, Z) that could potentially influence both patent activity and firm-specific risk. The inclusion of these theoretically motivated controls does not materially alter our core conclusions. The patent-volatility relationship maintains its statistical significance and economic magnitude across all augmented specifications, with coefficient estimates remaining within acceptable bounds of our baseline results. These findings strengthen confidence that our results reflect genuine patent-related risk effects rather than spurious correlations with unobserved firm characteristics.

## Discussion

This study provides novel empirical evidence on the relationship between patent activity and idiosyncratic volatility in the U.S. pharmaceutical industry, revealing complex dynamics that both support and challenge existing theoretical frameworks. Our findings demonstrate that patents play a significant stabilizing role in reducing firm-specific risk, with the coefficient indicating that increased patenting is associated with lower idiosyncratic volatility. This result aligns with the theoretical prediction that patents reduce market uncertainty by securing exclusive rights to commercialize innovations, thereby providing clearer expectations about future cash flows.

Our findings provide mixed support for existing theoretical frameworks in the innovation-finance literature. The core result that patents reduce idiosyncratic volatility aligns with the argument by Alshammasi and Almomen (6) that successful innovation outputs like patents can decrease firm-specific risks by reducing information uncertainty. This relationship supports the notion that patents function as stabilizing assets in uncertain industries, providing investors with tangible indicators of a firm’s technological capabilities and future revenue potential.

The progressive risk reduction observed as projects advance through development phases is consistent with findings by Mazzucato and Tancioni (7) that innovation-driven volatility tends to decrease as projects near commercialization. Our results extend this framework by providing granular evidence of how different stages of the pharmaceutical development process contribute to this risk evolution. The finding that Phase III projects and newly launched products most strongly reduce volatility supports the theoretical prediction that uncertainty diminishes as regulatory and technical risks are progressively resolved.

However, our findings contrast with some aspects of the framework proposed by Hirshleifer, Hsu (5), which suggested that innovation increases firm-specific risk due to market uncertainties about technological outcomes. The divergence in our results may stem from the specific characteristics of the pharmaceutical industry, where patent approvals often coincide with significant regulatory milestones that substantially reduce perceived risks. Unlike sectors where patents may not directly indicate market readiness, pharmaceutical patents serve as critical signals of a firm’s proximity to revenue generation, thereby exerting stronger stabilizing influences on investor expectations.

Our finding that patent expirations contribute to increased volatility provides empirical support for the theoretical framework outlined by Arroyabe (50), who discussed the heightened competitive pressures and loss of market exclusivity associated with patent cliffs. This phenomenon underscores the dual nature of patents in the pharmaceutical industry—while they provide protection and stability during their active period, their approaching expiration creates anticipatory uncertainty that manifests in increased firm-specific volatility. This result highlights the temporal dimension of patent value and suggests that effective patent portfolio management requires attention to both current protection and future expiration schedules.

While our results provide strong evidence for patent-related risk effects, several alternative explanations warrant consideration in interpreting these findings. Market sentiment toward the pharmaceutical sector represents one potential confounding factor, as changes in investor perceptions of the industry could drive fluctuations in idiosyncratic volatility independently of firm-specific patent activities. Periods of heightened optimism about drug development breakthroughs might reduce volatility across the sector, potentially coinciding with increased patent activity and creating spurious correlations.

The regulatory environment presents another important consideration, given the heavily regulated nature of pharmaceutical innovation. Changes in FDA approval processes, healthcare policies, or patent laws could significantly impact firm-specific risk while occurring concurrently with changes in patent activity. Our crisis-period analyses partially address this concern by demonstrating relationship stability across different regulatory and market environments, but future research could benefit from more explicit modeling of regulatory regime changes.

Firm-specific factors not fully captured in our control variables represent a third category of potential confounders. Management quality, R&D process efficiency, and overall innovation strategy might simultaneously influence both patent output and stock price stability. While we controlled for firm size and age, other less tangible organizational capabilities could be driving the observed relationships.

The broader economic environment provides a fourth potential explanation, as economic cycles might influence both innovation strategies and stock market volatility. During economic downturns, firms might reduce R&D spending and patent activity while market volatility generally increases, creating negative correlations that could be misinterpreted as causal relationships.

Finally, changes in investor behavior and market dynamics over our study period could alter how patent information is incorporated into stock prices. The evolution of investor sophistication, the proliferation of algorithmic trading, and changing risk appetites could all influence the observed relationship between patents and volatility, potentially affecting the stability of our findings across different time periods.

For investors, our findings highlight the importance of incorporating patent portfolio analysis into pharmaceutical investment strategies. The evidence that patents reduce idiosyncratic volatility suggests that firms with robust intellectual property positions may offer more stable investment opportunities, particularly valuable for risk-averse investors or those seeking to reduce portfolio volatility. However, investors should consider the temporal dynamics of patent protection, as approaching expirations may signal periods of increased uncertainty requiring careful portfolio adjustment.

The differential effects across development phases provide additional guidance for investment timing and risk assessment. Our results suggest that firms with substantial Phase III pipelines and recent product launches may offer lower firm-specific risk profiles, while those heavily concentrated in early-stage development may require higher risk premiums despite their growth potential.

Pharmaceutical managers can leverage these insights to optimize innovation strategies and communicate effectively with financial markets. The finding that advanced-stage projects reduce volatility suggests potential value in maintaining balanced development portfolios that combine high-risk early-stage research with more predictable late-stage development activities. This portfolio approach could help stabilize firm-specific risk while maintaining innovation pipeline flow.

The demonstrated relationship between patents and reduced volatility also suggests that effective intellectual property management extends beyond legal protection to encompass financial risk management. Companies might consider timing patent announcements and portfolio communications to coincide with periods of market uncertainty, potentially leveraging the stabilizing effects of patent information to maintain investor confidence.

Policymakers can draw on our findings to formulate regulatory frameworks that balance innovation incentives with market stability concerns. The evidence that patents reduce firm-specific risk supports the continuation of strong intellectual property protection as a mechanism for encouraging pharmaceutical innovation while maintaining stable investment environments. However, policymakers should also consider the potential market disruption associated with patent expirations and explore mechanisms to smooth these transitions.

The relationship between development phase advancement and risk reduction suggests that regulatory policies affecting clinical trial processes and approval timelines could have significant implications for market stability. Policies that provide greater predictability in regulatory processes might enhance the volatility-reducing effects of advanced-stage development activities, potentially improving capital allocation efficiency in pharmaceutical markets.

### Study limitations and boundary conditions

Our analysis is subject to several important limitations that define the boundaries of our conclusions. The geographic focus on U.S. pharmaceutical markets limits generalizability to other regulatory environments and patent systems, where different institutional frameworks might alter the patent-volatility relationship. The pharmaceutical industry’s unique characteristics—particularly its high R&D intensity, lengthy development cycles, and strong patent dependence—may not extend to other innovation-intensive sectors.

Our reliance on patent counts as innovation measures, while standard in the literature, does not account for heterogeneity in patent quality or impact. Some patents may have substantially greater market significance than others, potentially creating measurement error that could bias our estimates toward zero and understate the true economic relationships.

The econometric approach, while robust to many potential confounds, may not capture all complexities of market reactions to patent events. Factors such as investor sentiment, competitive dynamics, and broader industry trends could influence volatility patterns beyond the scope of our model specifications.

Finally, our sample period encompasses significant structural changes in both pharmaceutical markets and financial markets more broadly. While our crisis-period analyses suggest relationship stability, longer-term evolutionary changes in market structure, investor behavior, or regulatory frameworks could affect the sustainability of the patterns we document.

Despite these limitations, our findings provide robust evidence for the risk-mitigating role of patents in pharmaceutical markets and offer valuable insights for stakeholders navigating the complex intersection of innovation and financial risk in this critical industry.

## Conclusion

This study examines the relationship between patent activity and idiosyncratic volatility in the U.S. pharmaceutical industry, addressing a critical gap in the innovation-finance literature. Through comprehensive empirical analysis using the Fama-French 5-factor model and panel data spanning 2005–2024, we provide novel evidence on how intellectual property influences firm-specific risk in innovation-intensive markets.

Our findings establish that patents serve dual functions as both innovation protectors and financial risk stabilizers. Patent activity demonstrates a significant negative association with idiosyncratic volatility, supporting theoretical predictions that intellectual property reduces market uncertainty by providing clearer expectations about future cash flows. The analysis across pharmaceutical development phases reveals important temporal dynamics in the innovation-risk relationship, showing that while early-stage development activities introduce uncertainty, successful commercialization significantly reduces firm-specific volatility.

This research makes several important contributions to existing literature. It provides the first direct empirical evidence linking patent activity to firm-specific volatility in pharmaceuticals, introduces granular analysis of how different development stages affect risk over time, and reconciles conflicting theoretical perspectives on whether innovation increases or decreases firm-level uncertainty. The findings demonstrate that innovation initially elevates risk but ultimately stabilizes it through successful patent protection and market entry.

The implications extend to multiple stakeholder groups. Investors can incorporate patent portfolio considerations into risk assessment frameworks, managers can optimize innovation strategies to balance research potential with volatility management, and policymakers can design regulatory frameworks that support both innovation incentives and market stability. The demonstrated relationship between patent activity and risk reduction emphasizes the strategic importance of intellectual property management beyond traditional legal protection.

While the study’s focus on U.S. pharmaceutical markets and reliance on patent counts rather than quality measures present limitations, the findings provide robust empirical foundation for understanding innovation-finance dynamics. Future research should explore these relationships across different regulatory environments and industries to establish broader theoretical frameworks.

This study establishes patent activity as a fundamental determinant of firm-specific risk, offering valuable insights for stakeholders navigating the complex intersection of innovation and financial uncertainty in high-stakes industries where sustainable growth requires careful balance between research investment and market stability.
